# Supplementary material for: Photoelectrocatalytic Processes of TiO2 Film: The Dominating Factors for the Degradation of Methyl Orange and the Understanding of Mechanism
Source: Molecules. 2023 Dec 6;28(24):7967. doi: 10.3390/molecules28247967 (PMC10746121; doi:10.3390/molecules28247967)
Supplement: Supplementary file 1 [file molecules-28-07967-s001.zip › molecules-2726432-supplementary.pdf]

Article

Photoelectrocatalytic Processes of TiO_2 Film: The Dominating Factors for the Degradation of Methyl Orange and the Understanding of Mechanism

Yuhui Xiong [1,†], Sijie Ma [2,†], Xiaodong Hong [2], Jiapeng Long [1,*] and Guangjin Wang [2,*]

[1] School of Materials Science and Engineering, Shenyang University of Chemical Technology, Shenyang 110142, China; 2013020230@stu.syuct.edu.cn
[2] School of Materials Science and Hydrogen Energy, Foshan University, Foshan 528000, China; 15729508936@163.com (S.M.); hongxiaodong@lntu.edu.cn (X.H.)
* Correspondence: long2682@126.com (J.L.); wgj501@163.com (G.W.)
† These authors contributed equally to this work

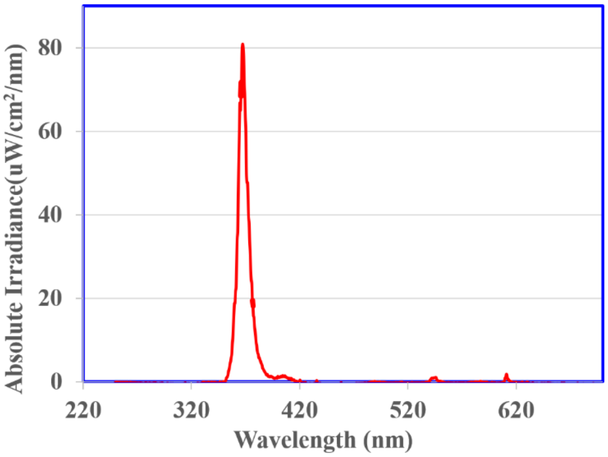

Figure S1. The light spectrum of the UV lamp used in irradiation experiments. The peak is located at 365 nm.

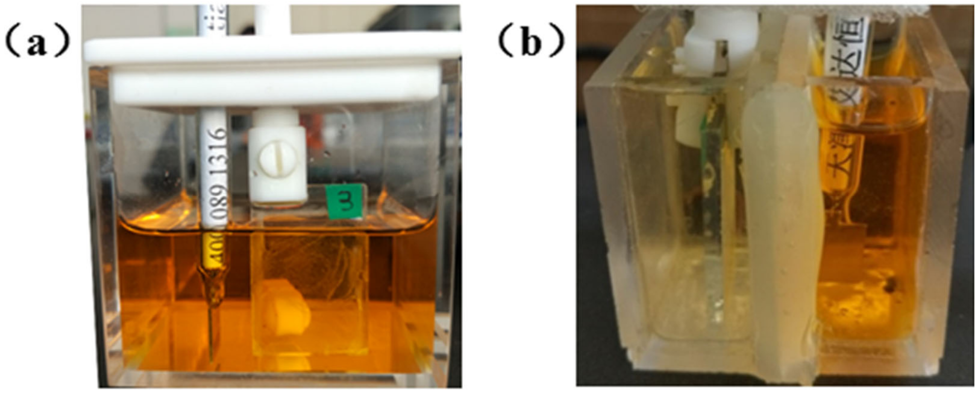

Figure S2. The photographs of experimental set-up of (a) PC, PEC reactors and (b) MS, PECMS reactors.

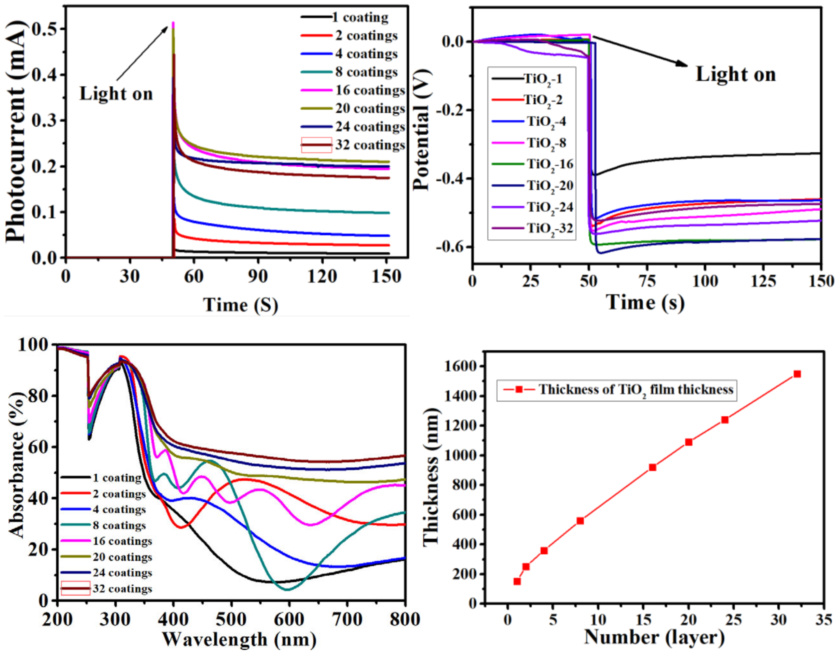

Figure S3. The short circuit photocurrent (a), open circuit potential (b) and (c) absorbance of TiO_2-1, -2,-4, -8, -16, -20, -24 and -32. (d) variation of film thickness as the layers of coatings.

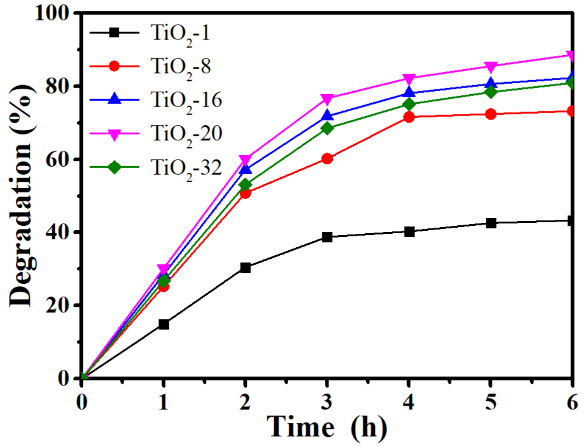

Figure S4. PEC degradation of MO byTiO_2-1, -8, -16,-20 and 32 photoelectrodes at 1.0 V bias under UV irradiation.
